# A mathematical model for active contraction in healthy and failing myocytes and left ventricles

**DOI:** 10.1371/journal.pone.0174834

**Published:** 2017-04-13

**Authors:** Li Cai, Yongheng Wang, Hao Gao, Yiqiang Li, Xiaoyu Luo

**Affiliations:** 1 NPU-UoG International Cooperative Lab for Computation & Application in Cardiology, Northwestern Polytechnical University, Xi’an, Shanxi Province, China; 2 School of Mathematics and Statistics, University of Glasgow, Glasgow, Scotland, United Kingdom; Georgia State University, UNITED STATES

## Abstract

Cardiovascular disease is one of the leading causes of death worldwide, in particular myocardial dysfunction, which may lead to heart failure eventually. Understanding the electro-mechanics of the heart will help in developing more effective clinical treatments. In this paper, we present a multi-scale electro-mechanics model of the left ventricle (LV). The Holzapfel-Ogden constitutive law was used to describe the passive myocardial response in tissue level, a modified Grandi-Pasqualini-Bers model was adopted to model calcium dynamics in individual myocytes, and the active tension was described using the Niederer-Hunter-Smith myofilament model. We first studied the electro-mechanics coupling in a single myocyte in the healthy and diseased left ventricle, and then the single cell model was embedded in a dynamic LV model to investigate the compensation mechanism of LV pump function due to myocardial dysfunction caused by abnormality in cellular calcium dynamics. The multi-scale LV model was solved using an in-house developed hybrid immersed boundary method with finite element extension. The predictions of the healthy LV model agreed well with the clinical measurements and other studies, and likewise, the results in the failing states were also consistent with clinical observations. In particular, we found that a low level of intracellular Ca^2+^ transient in myocytes can result in LV pump function failure even with increased myocardial contractility, decreased systolic blood pressure, and increased diastolic filling pressure, even though they will increase LV stroke volume. Our work suggested that treatments targeted at increased contractility and lowering the systolic blood pressure alone are not sufficient in preventing LV pump dysfunction, restoring a balanced physiological Ca^2+^ handling mechanism is necessary.

## Introduction

Myocardial dysfunction is a considerable social and economic burden because it can lead to heart failure due to repeated stresses and injuries [1]. However, our understanding of cardiac dysfunction remains incomplete. Mann and Bristow [2] suggested that heart failure could be viewed as a biomechanical model orchestrated by different subsystems, and the downstream biological remodelling is one of the drivers leading to myocardial dysfunction progression. Multi-scale and multi-physics biomechanical modelling of heart dynamics provides a unique way to gain insights of myocardial function and holds the potential for patient risk stratification and clinical decision making [3, 4].

Developing physiologically realistic heart models is complicated by many challenges [5], such as the complex anatomical geometry, nonlinear material responses of the myocardium, fluid-structure interaction, and issues across different length/function scales. In the last several decades, many nonlinear models of cardiac mechanics have been developed using the finite element (FE) method [6–9]. Different constitutive laws are introduced to characterize myocardium, such as the “pole-zero” constitutive law [9] and “Holzapfel-Ogden” law [10]. A recent review on heart modelling is provided in Ref [5]

In addition to the aforementioned structure-only ventricular models, immersed boundary (IB) method has also been used in modelling ventricular dynamics since the 1970s [11]. Recently, Griffith and Luo [12] have developed a hybrid IB with finite element method (IB/FE) that describes the immersed structure using the finite elements elasticity. Gao et al. [13] successfully applied the IB/FE framework to an imaging-derived subject-specific human LV model in diastole, as well as a diseased heart with myocardial infarction [14]. Later, Cai et al. [15] developed a more automatic procedure to construct mathematical models from medical images, and simulated the end-diastolic (ED) and end-systolic (ES) behaviours of a healthy left ventricle. Their results demonstrated that it is possible to simulate realistic ventricular dynamics using the IB/FE approach in conjunction with structure-based constitutive models [13]. However, the electrophysiological modelling of the heart was oversimplified in their models.

Myocardial contraction is highly dependent on the dynamics of calcium ion (Ca^2+^) in individual myocytes [16]. Numerious myocyte models exist in the literature [17], such as the model developed by Luo et al. [18], the FK model by Fenton et al. [19], and so on. Human ventricular myocyte models are emerging in recent years. For example, the ten Tusscher model [20], the OVVR model [21], and the Grandi-Pasqualini-Bers (GPB) model [22]. The electrophysiology models in single myocyte have proven to be very useful to study the mechanisms underlying disturbances of Ca^2+^ due to functional dysfunction [23] and the effects of drugs [24].

The failing heart undergoes continuous electrical-physiological, metabolic and structural remodelling in order to compensate the loss of functioning myocytes [2]. Ca^2+^ is the most important ion regulating myocardial excitation-contraction coupling [16]. Continuous remodelling of ion channels in failing myocyte, such as a significant increase in the late Na^+^ current [25], will cause AP duration prolongation and altered intracellular Ca^2+^ homeostasis, the hallmark characteristics of failing myocytes [26]. Several studies [27–29] have used the GPB model to explore excitation–contraction coupling and repolarization abnormalities in single human myocyte, in particular the Ca^2+^ handling process.

The multiscale nature of the heart requires one to model myocyte dynamics at cellular level and the electrophysiology at tissue level [17]. Many cardiac mechanical models, our previous models included, have not incorporated the detailed electrophysiology model for the myocytes. These models assumed a homogeneous and simultaneous contraction in the LV wall, and used prescribed intracellular calcium transient (CaT), or specified contraction not explicitly based on electrophysiology [7, 14, 30]. In this work, we coupled a more detailed model of myocyte ion dynamics for excitation-contraction coupling into the tissue-level mechanics to study how Ca^2+^ dynamics at the cellular level affects the myocardial function at organ level, in particular in failing heart. This was achieved by extending our IB/FE LV model [13, 14] by embedding a modified GPB model together with a myofilament model. The new coupled model was then used to study the myocardial active contraction at both cellular- and tissue- levels in healthy and failing states.

The remaining of the paper is organised as follows. In Methodology, we report how we use a modified GPB model to model the myocyte electrophysiology [29], and the filament model by Niederer et al. [31] (henceforth referred to as the NHS model) for the active contraction. This is followed by studying four different cases of myocyte contraction from healthy to failing states. In Results, we describe how we embed the active tension generation using the NHS and GPB model into a IB/FE LV model, to study the pump function in the healthy case and the worst failing case. We subsequently show how changes in cellular electrophysiology may affect the LV pump function at the organ level. The paper ends with Discussion and Conclusions.

## Methodology

### Myocyte dynamics at cellular level

#### Ion dynamics: The GPB model

In this study, a modified GPB model by Cardona et al. [29] was used to simulate the Ca^2+^ dynamics in healthy and failing states. The reasons for choosing the GPB model were that (1) it matches experimental data well [22]; (2) it is adequate to analyse action potential with detailed Ca^2+^ dynamics, which plays a crucial role in excitation-contraction in myocardium [16]; (3) other studies have demonstrated that it can reproduce the electrical changes of failing myocytes caused by remodelling in ion channels and transporters [28, 29]. The original GPB model consists of 38 ordinary differential equations, which describe the kinetics of different ion channels. Details of the GPB model and parameters are given in S1 and S2 Appendices.

In the failing myocytes, the intracellular sodium concentration and Ca^2+^ handing are closely related. For example, the intracellular sodium concentration, especially I_NaL_, is increased in failing myocytes, which contributes to cellular Ca^2+^ accumulation. The introduced I_NaL_ in the modified GBP model was modelled following the Hodgkin-Huxley formula [32],

$$I_{{\text{NaL}}_{\text{junc}}}=F_{\text{junc}}\;g_{\text{NaL}}\;m_{\text{L}}^{3}\;h_{\text{L}}\left(V_{\text{m}}-E_{\text{junc}}\right),$$ (1)

$$I_{{\text{NaL}}_{\text{sl}}}=F_{\text{sl}}\;g_{\text{NaL}}\;m_{\text{L}}^{3}\;h_{\text{L}}\left(V_{\text{m}}-E_{\text{sl}}\right),$$ (2)

$$I_{\text{NaL}}=I_{{\text{NaL}}_{\text{junc}}}+I_{{\text{NaL}}_{\text{sl}}},$$ (3)

and

$$\begin{array}{cc} \frac{d\;m_{\text{L}}}{dt} & =\alpha_{\text{m,L}}\;\left(1-m_{\text{L}}\right)-\beta_{\text{m,L}}\;m_{\text{L}},\\ \frac{d\;h_{\text{L}}}{dt} & =\frac{h_{\text{L},\infty }-h_{\text{L}}}{\tau_{\text{hl}}},\\ \alpha_{\text{m,L}} & =\frac{0.32(V_{\text{m}}+47.13)}{1-e^{-0.1(V_{\text{m}}+47.13)}},\\ \beta_{\text{m,L}} & =0.08e^{-\frac{V_{\text{m}}}{11}},\\ h_{\text{L},\infty } & =\frac{1}{1-e^{\frac{V_{\text{m}}+91}{6.1}}}, \end{array}$$

where g_Nal_ is the maximum conductance, m_L_ is the activation gate, h_L_ is the steady-state inactivation gate, *τ*_hl_ (233 ms) is the time constant of inactivation, E_junc_ and E_sl_ are, respectively, the Nernst potential for the sodium ion in the junctional and subsarcolemmal compartments (see S1 and S2 Appendices for details). Cardona et al. [29] found that the most impactful parameters on Ca^2+^ handing are the sarcoplasmic reticulum Ca-ATPase (SERCA) function, I_NaL_, the Na/K pump current (I_Nak_), the sarcoplasmic reticulum leak current (I_leak_), the background Ca current (I_Cab_) and the Na-Ca exchanger current (I_ncx_). The modified GPB model was further extended by Trenor et al. [28], who introduced the impact factors to simulate the transition from healthy to failing states. The impact factors are coefficients that can change the parameters on Ca^2+^ handling in the modified GPB model. The final formulations of the modified GPB model employed in this study are given in S3 Appendix.

Based on the work of Trenor et al. [28], four cases were designed to represent different failing states; Case A: the healthy state, Cases B and C: two intermediate failing states, and Case D: the end-stage failure state. The impact factors of Case A and Case D were adopted from [28], and the impact factors of Case B and Case C were obtained by reducing the values of Case A by one-third and two-thirds of the difference between Cases A and D, as shown in Table 1. In healthy state, the peak value of the intracellular CaT obtained by this modified GPB model were between 0.3 *μ*M and 0.4 *μ*M. Thus, in order to make the peak CaT to be around 1 *μ*M which was needed by the later active contraction model [31], two diffusion constants were changed (needing to note that changes of ${\text{J}}_{\text{Ca}}{}_{{}_{\text{juncsl}}}$ and ${\text{J}}_{\text{Ca}}{}_{{}_{\text{slmyo}}}$ may not be the ideal way to obtain an intracellular CaT profile with a peak value of 1 *μ*M), they were (1) the diffusion constant between the junctional cleft and subscarcolemmal: ${\text{J}}_{\text{Ca}}{}_{{}_{\text{juncsl}}}\;=\;1.2362\;\times \;10^{-12}$ L/ms, and (2) the diffusion constant between the subsarcolemmal and the bulk cytosol space: ${\text{J}}_{\text{Ca}}{}_{{}_{\text{slmyo}}}\;=\;7.4485\;\times \;10^{-12}$ L/ms.

**Table 1:** Impact factors in the four different cases based on the modified GPB model.

	*f*_hl_	*f*_NaL_	*f*_Ki_	*f*_NaK_	*f*_Nabk_	*f*_Cabk_	*f*_ncx_	*f*_to_	*f*_SRleak_	*f*_SRca_	*f*_EC50SR_
Case A	1	1	1	1	1	1	1	1	1	1	1
Case B	1.333	1.333	0.893	0.833	0.667	1.177	1.25	0.8	2.333	0.833	0.963
Case C	1.666	1.666	0.787	0.667	0.333	1.353	1.5	0.6	3.667	0.667	0.927
Case D	2	2	0.68	0.5	0	1.530	1.75	0.4	5	0.5	0.89
impact factor	the relative physical quantity of the impact factor										
*f*_hl_	the time constant of inactivation(*τ*_hl_)										
*f*_NaL_	the late sodion current (I_NaL_)										
*f*_ki_	the inward rectifier K current (I_Ki_)										
*f*_NaK_	the Na/K pump current (I_NaK_)										
*f*_Nabk_	the background Na current (I_Nabk_)										
*f*_Cabk_	the background Ca current (I_Cabk_)										
*f*_ncx_	the Na-Ca exchanger current (I_ncx_)										
*f*_SRleak_	the current density of the sarcoplasmic reticulum leak current (J_SR_leak__)										
*f*_to_	the transient outward K current (I_to_)										
*f*_SRrca_	the current density of the sarcoplasmic reticulum Ca-ATPase (J_serca_)										
*f*_EC50SR_	the half maximal effective concentration in sarcoplasmic reticulum (EC50SR)										

#### Myofilament active contraction: The NHS model

The active tension in each myocyte, *T*(*t*), was modelled as a function of the myocyte stretch (λ_*f*_), the stretch rate ($\frac{d\lambda_{f}}{dt}$), and the intracellular CaT [31]. Details of the NHS model and the parameters are given in S4 Appendix. To study the influence of CaT on the active tension generation in individual myocytes, the intracellular CaT from the modified GPB model was coupled to the NHS model to trigger active contraction. We further assumed that in the initial time *T* = 0 kPa, and λ_*f*_ = 1, $\frac{d\lambda_{f}}{dt}=0$ during myocyte contraction. Therefore, the simulated active tension in one myocyte corresponded to an isometric tension experiment at resting length.

### Myocardial dynamics at organ level: The LV model

#### IB/FE formulation

The in-house developed IB/FE framework was employed to simulate the LV contraction [12], in which the Lagrangian equations of the LV dynamics were approximated on an FE mesh, and the Eulerian equations of the fluid were approximated on a Cartesian grid [11]. A regularised delta function *δ* was used to describe the fluid-structure interaction, which implies that the IB/FE approach permits nonconforming discretization of the fluid and structure domains. In brief, the governing equations of the coupled fluid-structure system are

$$\rho \left(\frac{\partial u(x,t)}{\partial t}+u\left(x,t\right)\cdot \nabla u\left(x,t\right)\right)=-\nabla p\left(x,t\right)+\mu \nabla^{2}u\left(x,t\right)+f^{\text{e}}\left(x,t\right),$$ (4)

**Fig 1:**
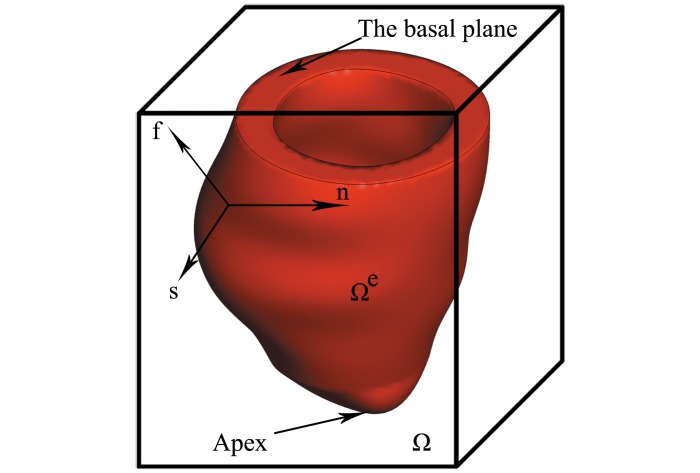
Schematic illustration of the LV model. Ω is the total computational domain, Ω^*e*^ is the LV structure, **f** denotes the myofibre direction, **s** denotes the sheet direction, and **n** denotes the normal orientation of the plane spanned by the myofibre and sheet directions.

∇·u(x,t)=0, (5)

$$f^{\text{e}}\left(x,t\right)=\int_{E}F^{e}\left(X,t\right)\delta \left(x-\chi \left(X,t\right)\right)dX,$$ (6)

$$\frac{\partial \chi (X,t)}{\partial t}=\int_{\Omega }u(x,t)\delta (x-\chi (X,t))dfx,$$ (7)

where Ω⊂*R*^3^ denotes the physical region occupied by the fluid-structure system, and *E*⊂*R*^3^ denotes the region occupied by the immersed solid in the reference configuration. *ρ* is the fluid density, *p* is the pressure, and *μ* is the viscosity. **x** = (*x*_1_, *x*_2_, *x*_3_) ∈ Ω denotes the Cartesian (Eulerian) coordinates, **X** = (*X*_1_, *X*_2_, *X*_3_) ∈ *E* denotes the material (Lagrangian) coordinates in the reference configuration. ***χ***(**X**, *t*) ∈ Ω gives the physical position of material point **X** at time *t*. Therefore, the physical region occupied by the structure at time *t* is Ω^*e*^(*t*) = ***χ***(*E*, *t*), and the physical domain occupied by the fluid at time *t* is Ω^*f*^ (*t*) = Ω − Ω^*e*^(*t*). The IB/FE LV model is shown in Fig 1.

The total Cauchy stress tensor of the coupled fluid-structure system was defined as

$$\sigma \left(x,t\right)=\sigma^{\text{f}}+\left\{\begin{array}{cc} \sigma^{\text{e}} & \text{for}\;x\in \Omega^{e},\\ 0 & \text{for}\;x\in \Omega /\Omega^{e}, \end{array}\right.$$ (8)

where ***σ***^f^ = −*p***I** + *μ*[∇**u** + ∇**u^T^**] is the stress tensor of a viscous incompressible fluid, existing in both the solid and fluid regions. **I** is the identity matrix, and ***σ***^e^ is the elastic stress tensor determined by the deformation of the immersed structure, defined as

$$\sigma^{\text{e}}=\left\{\begin{array}{cc} J^{-1}P^{\text{e}}F^{\text{e}} & \text{for}\;x\in \Omega^{\text{e}},\\ 0 & \text{otherwise}, \end{array}\right.$$ (9)

where $F=\frac{\partial \chi }{\partial X}$ is the structural deformation gradient and *J* = det(**F**), **P**^e^ is the first Piola-Kirchoff stress tensor, consisting of the passive elastic response of the myocardium **P**^p^ and the active stress **P**^a^, that is

$$P^{\text{e}}=P^{\text{p}}+P^{\text{a}}.$$ (10)

**P**^p^ was calculated from a strain-invariant based strain-energy function (W) introduced by Holzapfel and Ogden [10],

$$W=\frac{a}{2b}\;\exp^{b(I_{1}-3)}+\sum_{i=f,s}\frac{a_{i}}{2b_{i}}\;\left(\exp^{b_{i}{\left(I_{4i}^{\prime }-1\right)}^{2}}-1\right)+\frac{a_{\text{fs}}}{2b_{\text{fs}}}\left(\exp^{\left(b_{\text{fs}}{\left(I_{\text{8fs}}\right)}^{2}\right)}-1\right),$$ (11)

and

$$P^{\text{p}}=\frac{\partial W}{\partial F}-a\;\exp^{b(I_{1}-3)}F^{-T}\;\left(x\in \Omega^{e}\right),$$ (12)

where *a*, *b*, *a*_*i*_ and *b*_*i*_ (*i* = f, s, fs) are eight non-negative material parameters. *I*_1_ = tr(**C**), and **C** = **F**^*T*^ **F** is the right Cauchy-Green deformation tensor. $I_{4i}^{\prime }=\max\left(I_{4i},1\right)$ (*i* = f, s), $I_{\text{4f}}=f_{0}^{T}Cf_{0}=f\cdot f$, **f** = **Ff**_0_, and $I_{\text{4s}}=s_{0}^{T}Cs_{0}=s\cdot s$, **s** = **Fs**_0_. **f**_0_ and **s**_0_ are the fibre and sheet directions in the reference configuration, as shown in Fig 1. *I*_4f_ and *I*_4s_ are the squared stretches along the fibre and sheet directions, respectively. $I_{4i}^{\prime }$ ensures that the myofibres only exert stresses when stretched. The coupling effects of the fibre and sheet directions are represented by $I_{\text{8fs}}=f_{0}^{T}Cs_{0}$. The last term in Eq (12) is to ensure that when **F** = **I**, **P**^p^ = 0.

The active contraction stress tensor in the current configuration was

$$\sigma^{\text{a}}=T\left(x,t\right)\bar{f}⊗\bar{f},$$ (13)

where *T*(**x**, *t*) is the active tension based on the NHS model, triggered by the intracellular CaT from the modified GPB model, $\bar{f}$ is the unit vector of the current myofibre direction. The NHS model parameters were initially obtained from animal experiments. To apply the NHS model to human heart, we introduced an extra scaling parameter *T*_scale_ in Eq (13) as in our previous study [14],

$$\sigma^{\text{a}}=T_{\text{scale}}\;T\left(x,t\right)\bar{f}⊗\bar{f}.$$ (14)

#### LV model construction and implementation

The LV model was constructed from an in vivo magnetic resonance imaging study of a healthy volunteer [15, 33], and a rule-based method was used to generate the myocardial fibre and sheet directions [8]. In the simulations, the physical domain occupied by the fluid-structure system (Ω) was taken to be a 15 cm×15 cm×20 cm box that was discretized with 96 × 96 × 128 Cartesian grids. An explicit version of crank Nicolson-Adams Bashforth scheme was used for time stepping, which required a relatively small time step size (1.22 × 10^−4^s). The LV model was implemented within the open-source IBAMR software framework (https://github.com/IBAMR/IBAMR).

The boundary conditions used were such that the longitudinal and circumferential displacements of the basal plane were constrained, whereas the radial displacements of the basal plane were set free. The no-slip condition was imposed on the left ventricular wall, and a zero pressure condition was applied on the physical domain of the fluid boundary. A spatially uniform pressure was loaded on the endocardial surface. Because it is difficult to measure the LV pressure in vivo, a population-based ED pressure (8 mmHg) was assumed [15]. In diastolic filling, the endocardial pressure was linearly increased from 0 to the assumed ED pressure. Then the myocardium started to contract with increased intracellular CaT and increased systolic pressure. To simulate the ES state, both the ES pressure (150 mmHg, obtained from the cuff measurements of the subject) and the peak value of intracellular CaT were maintained until a steady-state LV dynamics was reached.

The parameters used in the IB/FE LV model were: *a* = 0.24 kPa, *b* = 5.08, *a*_f_ = 1.46 kPa, *b*_f_ = 4.15, *a*_s_ = 0.87 kPa, *b*_s_ = 1.6, *a*_fs_ = 0.3 kPa, *b*_fs_ = 1.3 and *T*_scale_ = 3.0. These were obtained inversely by ensuring that the LV volume differences between the clinical measurements and the simulated values were less than 5% at end-diastole and end-systole [14].

## Results

### Myocyte contraction

**Fig 2:**
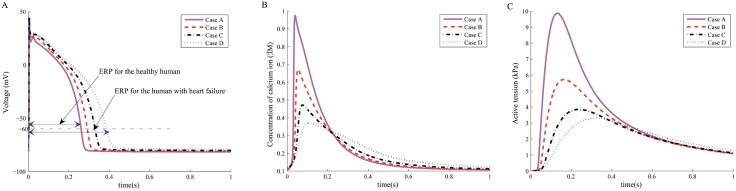
Numerical results of myocyte contraction in a single myocyte. Profiles of (A) action potential, (B) Intracellular CaT, and (C) Active tension.

The simulated AP profiles in individual myocytes in one cardiac cycle for the fours cases are showed in Fig 2(A), in which the spike notch dome morphology can be found. The characteristics of the AP profiles are summarised in Table 2. In Case A, the AP amplitude is 122.33 mV, which is comparable to published experimental measurements (100 mV [34], 132 mV [35], and 135 mV [36]). The maximal upstroke velocity in Case A is also within the range of experimental measurements (228 ± 11 V/s [37] to 446 ± 46 V/s [38]). The APD in Case D is much longer than that in Case A, similarly for the effective refractory period (ERP, measured as the interval between the AP upstroke and the repolarization level). The resting potential, the amplitude of the AP, and the maximal upstroke velocity in Cases B, C and D are much lower than the values in Case A.

**Table 2:** Characteristics of the active potential profiles, the intracellular CaT, and the active tension generation in a single myocyte.

	Case A	Case B	Case C	Case D
**Action Potential**				
APD_90_ (ms)	270.19	301.91	341.77	399.89
Resting potential (mV)	-80.55	-80.00	-79.17	-77.86
amplitude (mV)	123.36	123.87	123.86	122.42
maximal upstroke velocity (V/s)	370.70	354.71	330.71	291.18
ERP (ms)	261.06	291.23	330.00	385.89
**Intracellular CaT**				
Peak (*μ*M)	0.9752	0.6712	0.4714	0.3690
Time to peak of CaT (s)	0.039	0.054	0.076	0.106
Maximal upstroke velocity (*μ*M/s)	139.54	45.97	14.19	5.10
CaT amplitude (*μ*M)	0.87	0.56	0.36	0.25
**Active Tension**				
Peak (kPa)	9.87	5.72	3.85	3.31
Rate of tension development (kPa/s)	211.96	99.20	43.62	22.98
Time from peak tension to 50% relaxation (RT50) (s)	0.182	0.294	0.412	0.497

The comparison of intracellular CaT in the four cases are shown in Fig 2(B), and their characteristics in Table 2. The intracellular CaT in Case A peaks rapidly with the highest peak value compared to other cases. In contrast, the CaT in Case D peaks tardily at a much lower value, almost 63% less than the value in Case A. The intracellular CaT from the two intermediate cases (Case B and Case C) lie between Case A and Case D. After 0.2s until 0.6s, the CaT in Case D is slightly higher than others (Fig 2(B)). The maximal upstroke velocity is also highest in Case A and lowest in Case D.

Fig 2(C) shows the isometric active tension generation for the four cases, and the characteristics of the generated active tension are also summarized in Table 2. It can be found that the generated active tension and the maximal upstroke velocity decrease with the decrease of the intracellular CaT. In Cases C and D, the peak active tension is about one-third of the value in Case A, with a much lower upstroke velocity. Interestingly, the developed active tension in Case D after 0.5s is slightly higher than other three cases, which could partially be explained by the higher CaT in Case D after 0.2s as shown in Fig 2(B), however, the difference in active tension is not much.

### LV contraction

**Fig 3:**
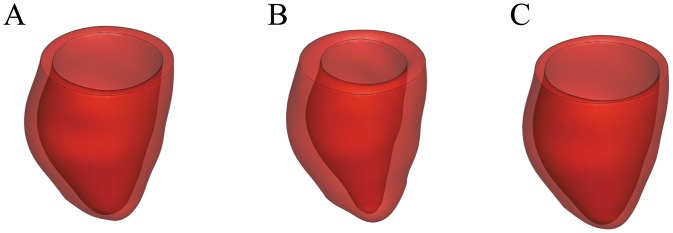
Deformed LV structures. A: Deformed LV structures at end-diastole; B: the end-systolic state for Case A; C: the end-systolic state for Case D.

The deformed end-diastolic LV geometries are same in Cases A and D because of the same passive material properties and boundary conditions used, shown in Fig 3(A). The deformed end-systolic geometry in Case A is shown in Fig 3(B). In Case A, the LV cavity volume is 145 mL at end-diastole and 61 mL at end-systole, with an ejection fraction (EF) of 57.55%, which are in agreement with the measured end-diastolic and end-systolic volumes (ED: 143 mL, ES: 64 mL). The deformed end-systolic geometry in Case D is showed in Fig 3(C), the corresponding LV cavity volume is 189 mL, and the mean myofibre strain is 0.07 ± 0.06. The larger end-systolic LV cavity volume and the positive systolic myofibre strain in Case D suggest that the LV model does not contract. This is largely because of the much lower level of intracellular CaT in Case D, which results in a much lower systolic active tension. The average systolic active tension in Case D is 50.2 ± 4.3 kPa, a 38% less than that of Case A (81.53 ± 21.0 kPa). The lower myocardial active tension in Case D agrees with the cellular level results: the isometric tension in Case D is 66% less compared to the peak value in Case A.

**Fig 4:**
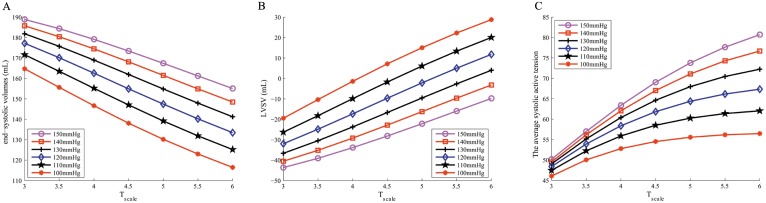
LV pump function in case D under different end-systolic pressures and *T*_scale_. A: The end-systolic volume; B: LV stroke volume; C: Average systolic active tension.

Remodelling in myocytes, such as increasing myocardial contractility (i.e *T*_scale_), decreasing the afterload (i.e. the end-systolic pressure), or increasing diastolic filling pressure (i.e. the ED pressure), is needed to maintain the minimum required pump function in a failing heart [2, 39]. We further investigated the effects of decreasing the end-systolic pressure and increasing *T*_scale_ on LV pump function in Case D, and the results are shown in Fig 4(A). As expected, the LV end-systolic volume decreases with the decrease of the end-systolic pressure or the increase of *T*_scale_. Fig 4(B) shows the corresponding changes of LV stroke volume, defined as ED volume—ES volume. When the end-systolic pressure is 100 mmHg and *T*_scale_ is 6.0, the end-systolic volume is 116 mL and an EF of 19% is achieved in Case D, which still indicates a severe heart failure. The LV stroke volume in case A is 83.60 mL, almost four times greater. Fig 4(C) is the average systolic active tension in Case D. For a given end-systolic pressure, a higher *T*_scale_ is associated with a higher active tension and a stronger contraction. On the other hand, for a given *T*_scale_, although a lower end-systolic pressure reduces the active tension generation, the LV stroke volume actually increases. This is because the active tension is length and strain-rate dependent, therefore a lower end-systolic pressure means that the myocardium is able to contract early and quickly, leading to a larger LV stroke volume. Fig 4 shows that only when the end-systolic pressure is below 120 mmHg, the LV can pump the blood into the systemic circulation. These results may suggest that it is insufficient to improve the pump performance by simply increasing the myocardial contraction, or reducing the end-systolic pressure.

**Fig 5:**
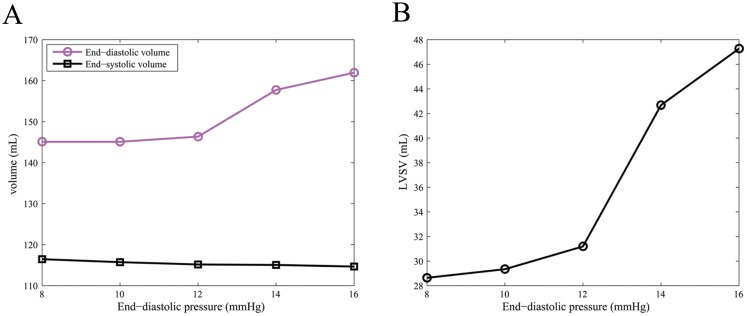
LV pump function under different end-diastolic pressures in case D. A: The end-diastolic and end-systolic volumes; B: LV stroke volume.

Fig 5(A) shows the end-diastolic and end-systolic volumes under different end-diastolic pressures in case D for a given end-systolic pressure (= 100 mmHg) and *T*_scale_(= 6.0). The end-diastolic volume increases with the increase of the end-diastolic pressure, which is consistent with the Frank-Starling law of the heart. On the other hand, the end-systolic volume does not change much. The LV stroke volume versus end-diastolic pressure is shown in Fig 5(B). The LV stroke volume and EF reach their maximum values (47mL, 29.19%) at end-diastolic pressure = 16 mmHg, but still less than the value in Case A. The results suggest that with increased end-diastolic pressure, the left ventricle can pump more blood, which is beneficial in terms of meeting body’s blood demand. However, studies have found that elevated diastolic filling pressure may associate with terminal heart failure in the longer run [40, 41].

## Discussion

From biomechanical perspective, myocaridal dysfunction could be considered as biomechanical model with interrelated and intricate changes and remodelling in cardiac structure and function as the result of downstream biological abnormalities [2]. Advanced biomechanical modelling of heart mechanics with multi-scale and multi-physics may hold the potential to improve our understanding of failing hearts and shed lights on effective treatments [3]. This computational work, based on an advance human myocyte electrophysiological model, a myofilament model for excitation-contraction coupling, and a multi-physics organ-level LV mechanics model, demonstrates that a multi-scale biomechanical model can be used to investigate the effects of downstream biological abnormalities on ventricular pump function. Once validated, it will provide a platform to understand the functional and structural remodelling in failing hearts.

Using this multi-scale LV model, we found that (1) profiles of AP, intracellular CaT and active tension are very different at cellular level from healthy to failing states; (2) a lower CaT can lead to a much less active tension generation; and (3) a lower intracellular CaT in individual myocytes can result in LV pump function failure even with increased myocardial contractility, decreased afterload, and increased diastolic filling pressure. Our modelling results at both the cellular and tissue levels for one healthy and diseased LV cases showed good agreement with experimental measurements and earlier studies, additional comparison is provided in S1 Table.

Specifically, the APD in the failing case (D) is prolonged compared to the value in the healthy case (A) because of the much slower rate of repolarization in Case D, this is consistent with experimental findings [42, 43]. As the myocardial dysfunction worsens (from Case A to Case D), the peak and the maximal upstroke velocity of intracellular CaT decreases, and the curve gradually flattens. With decreased intracellular CaT and its upstroke velocity, the generated active tension also decreases, to almost 66% in Case D. Although the CaT in Case D is higher than other cases after 0.2s, the active tension in Case D is generally lower than in Case A. This suggests that in a failing state, the remodelling in ion dynamics (by prolonging APD and increasing CaT in the later contraction phase) has limited effect on active tension generation. Therefore, our study suggests that a well-balanced physiological Ca^2+^ handling mechanism is an essential component in maintaining normal myocyte contractile function, which agrees with [44].

By simulating a pseudo-isometric experiment in a myocyte, we found that the active tension from Case D is much lower compared to Case A. To see how this affects the LV performance, we embedded the modified GPB model into a dynamic LV model [14]. Our results show that the reduction in average systolic active tension across the whole LV in Case D is 39% compared to the Case A, and no blood can be pumped out (the LV cavity volume at end-systole is greater than its end-diastolic volume). This is when sudden death occurs.

A decline in LV pump function will activate various compensatory mechanisms to restore a normal homeostatic cardiovascular function [2, 39]. These include a higher heart rate, myocyte hypertrophy, and increased LV end-diastolic volume (i.e. through elevated diastolic filling pressure). Treatments of heart failure typically aims to preserve the cardiac output by reducing the blood pressure (the afterload) using intravenous vasodilators, or increasing the myocardial contractility using positive iontropes [45, 46]. Indeed, our results show that decreased end-systolic pressure and increased myocardial contractility are not enough to maintain normal LV pump function. For instance, with the end-systolic pressure of 100 mmHg, the LV model can pump the blood out only when *T*_scale_ is above 4. However, the pump function is compromised even when *T*_scale_ is increased to 6, the LV stroke volume is only 29 mL, which is far less compared to the healthy Case A (83 mL). Moreover, continuous increase of the myocardial contractility can deteriorate the LV function in the longer term leading to cardiac de-compensation. It is perhaps for this reason that some inotropic therapies increased, rather than decreased, mortality [47].

In addition, the capacity of decreasing afterload or increasing myocardial contractility is not without limitation, therefore, other compensation mechanisms will be activated, such as elevated diastolic filling pressure through the Frank-Starling law of the heart [39, 40]. In case D, when the end-systolic pressure is 100 mmHg and *T*_scale_ is 6, the LV stroke volume increases with the increase of end-diastolic pressure. The LV stroke volume reaches 48 mL when the end-diastolic pressure is 16 mmHg. It is expected that with further increased end-diastolic pressure, LV stroke volume will increase further. However, clinical observations suggest that the elevated diastolic filling pressure would reflect patients at increased risk of developing late clinical symptoms of heart failure in the long term [41], even though it can increase the LV stroke volume in the short term as suggested by our models.

There are two recent mathematical descriptions of human ventricle myocytes, the GPB model [22] and the OVVR model [21]. Both models can match experimental data well though discrepancies exist in cellular and tissue levels [27, 48]. In this study the GPB model was chosen because it is adequate to analyse action potential, Ca^2+^ dynamics. Furthermore, the GPB model has been demonstrated to reproduce the electrical changes of failing myocytes due to ion channel and transporter remodelling [28, 48]. However, the issue with linking the GPB model to the NHS model is that the peak CaT in the GPB model is less than 1*μ*M, which is not compatible with the active tension generation model in the multi-scale LV model. Thus, we had to increase two diffusion constants so that the peak CaT is around 1*μ*M. OVVR model, on the other hand, has a CaT amplitude of about 1*μ*M. The adjustments of J_Cajuncsl_ and J_Caslmyo_ may not be the ideal way to tune the GPB model to obtain the required CaT profile, a carefully designed calibration procedure is required. Alternatively, one can use a different myofilament model to make the two models compatible since the NHS model was developed based on animal experiments. Despite of this, our modelling results from the adopted GPB model agree well with prior studies and experiments, for example, in the failing state, the CaT amplitude decreases, its rise and decay rates are slowed down [49], additional comparisons with other studies are provided in a supplementary table. We expect it will work equally well by replacing the GPB model with the OVVR model in our model, and it will be interesting to compare the behaviour of both models in a multi-scale biomechanical myocardial model, and explore other myofilament models developed for human.

The remodelling of the failing heart occurs in many aspects at both cellular and organ levels, in terms of structure [50], electrophysiology [51], metabolism [52], and couplings among them [16]. Cardiomyocytes in healthy and failing hearts differ in numerous ways, such as prolongation of AP, altered Ca^2+^ handing, intracellular Na^+^ accumulation, and impaired electro-mechanical coupling [16, 44, 51]. It is well known that failing myocytes have abnormal CaT, with decreased amplitude and slowed kinetics [49]. Except for the functional remodelling of ion channels and pumps as described in the adopted GPB model, cellular structural remodelling is also recognized as one of the underlying causes for altered intracellular Ca^2+^ homeostasis. These include alternations in the T-tubule structure [53], micro-architecture changes in sarcoplasmic reticulum [54], and alterations in molecular and biochemical structure of myofibrils [55]. Furthermore, metabolic remodelling also contributes to the impaired excitation-contraction coupling due to ATP deficiency [52]. In this study, we only studied the effects of CaT from different failing stages on myocardial active contraction that is caused by ion channel and transporter remodelling [28, 48]. However, we expect that other types of remodelling mechanism can be ready implemented in our model by including more detailed descriptions of related remodelling process.

Similar as the GPB model used here, the structural remodelling of the myocyte is not modelled in the NHS model [31]. An anatomical multi-scale myocardial description from sub-cellular to organ levels is needed in order to capture the spatially varied structural remodelling. However, there are many challenges in following this approach [56]. Even if one can develop detailed mathematical description based on proper upscaling, to solve this a model also requires efficient numerical methods and high-performance computers.

Other limitations of the work include: (1) we have not thoroughly analysed the effects of individual ion channel remodelling on myocardial active contraction, but focused on how CaT profiles from different failing stages affect LV pump function; (2) we assumed that the LV contraction was spatially homogeneous and temporally simultaneous, which has been widely adopted in heart simulations [6]. This treatment has limitations that the LV model will not be applicable to ischemic myocardial dysfunction, i.e. myocardial infarction, but more relevant to nonischemics myocardial dysfunction, such as ventricular fibrosis; (3) we also only simulated the end-diastolic and end-systolic phases of LV dynamics, not the whole cardiac cycle which involves interactions of the heart valves. Therefore, the developed multi-scale myocardial contraction model should be considered as a phenotype representation of failing hearts, rather than a personalized model. Nevertheless, this computational work provides a multi-scale platform for studying the effects of myocardial remodelling on LV pump function in a biomechanical perspective.

## Conclusion

In this study, we have modelled myocardial excitation-contraction coupling both at cellular and organ levels by incorporating a modified GPB model and the NHS model at healthy and failing states. The LV model was implemented in an in-house developed IB/FE framework. Results show that the active tension decreases with the decrease of intracellular CaT both at cellular and organ levels. In a failing heart, decreased systolic blood pressure, enhanced contractility and elevated diastolic filling pressure can improve heart pump function in the short term (i.e. increased stroke volume), however, they may lead to terminal heart failure in a longer term if a balanced physiological calcium ion handling mechanism is not restored. This study forms part of the continuous effort in multi-scale electro-mechanics modelling for clinical problems, and provides a platform to study remodelling in a failing heart from a biomechanical perspective.

## Supporting information

S1 Appendix Model equations of the original GPB model. (PDF)

S2 Appendix Parameters of the original GPB model. (PDF)

S3 Appendix The modified GPB model. The modified GPB model is formulated by introducing the impact factors (see Table 1) to the original GPB model.
(PDF)

S4 Appendix The NHS myofilament model. (PDF)

S1 Table Comparisons between the healthy cases and other studies. Comparisons are made between the modelling results from healthy cases and published experimental and numerical studies at organ and cellular level.
(PDF)
