# Supplementary material for: A mathematical model for active contraction in healthy and failing myocytes and left ventricles
Source: PLoS One. 2017 Apr 13;12(4):e0174834. doi: 10.1371/journal.pone.0174834 (PMC5391010; doi:10.1371/journal.pone.0174834)
Supplement: S1 Appendix — (PDF) [file pone.0174834.s001.pdf]

S1 Appendix: Model equations of the original GPB model

I_{Na}: Fast Na Current

$$m_{ss} = \frac{1}{(1+e^{-\frac{56.86+V_m}{9.03}})^2} \quad (S1.1)$$

$$\tau_m = 0.1292e^{-(\frac{V_m+45.79}{15.54})^2} + 0.06487e^{-(\frac{V_m-4.823}{51.12})^2} \quad (S1.2)$$

$$a_h = \begin{cases} 0.057e^{-\frac{V_m+80}{6.8}} & V_m < -40mV \\ 0 & \text{otherwise} \end{cases} \quad (S1.3)$$

$$b_h = \begin{cases} 2.7e^{0.079V_m} + 3.1 \times 10^5 e^{0.3485V_m} & V_m < -40mV \\ \frac{0.77}{0.13(1+e^{-\frac{V_m+10.66}{11.1}})} & \text{otherwise} \end{cases} \quad (S1.4)$$

$$\tau_h = \frac{1}{a_h+b_h} \quad (S1.5)$$

$$h_{ss} = \frac{1}{(1+e^{\frac{V_m+71.55}{7.43}})^2} \quad (S1.6)$$

$$a_j = \begin{cases} \frac{-2.5428\times10^4 e^{0.2444V_m} - 6.94810\times10^{-6} e^{-0.04391V_m}(V_m+37.78)}{1+e^{0.311(V_m+79.23)}} & V_m < -40mV \\ 0 & \text{otherwise} \end{cases} \quad (S1.7)$$

$$b_j = \begin{cases} \frac{0.02424e^{-0.01052V_m}}{1+e^{-0.1378(V_m+40.14)}} & V_m < -40mV \\ \frac{0.6e^{0.057V_m}}{1+e^{-0.1(V_m+32)}} & \text{otherwise} \end{cases} \quad (S1.8)$$

$$\tau_j = \frac{1}{a_j+b_j} \quad (S1.9)$$

$$j_{ss} = \frac{1}{(1+e^{\frac{V_m+71.55}{7.43}})^2} \quad (S1.10)$$

$$\dot{m} = \frac{m_{ss}-m}{\tau_m} \quad (S1.11)$$

$$\dot{h} = \frac{h_{ss}-h}{\tau_h} \quad (S1.12)$$

$$\dot{j} = \frac{j_{ss}-j}{\tau_j} \quad (S1.13)$$

$$I_{Na_{junc}} = F_{junc} \cdot G_{Na} \cdot m^3 \cdot h \cdot j \cdot (V_m - E_{Na_{junc}}) \quad (S1.14)$$

$$I_{Na_{sl}} = F_{sl} \cdot G_{Na} \cdot m^3 \cdot h \cdot j \cdot (V_m - E_{Na_{sl}}) \quad (S1.15)$$

$$I_{Na} = I_{Na_{junc}} + I_{Na_{sl}} \quad (S1.16)$$

I_{Nabk}: Background Na current

$$I_{Nabk_{junc}} = F_{junc} \cdot G_{NaB} \cdot (V_m - E_{Na_{junc}}) \quad (S1.17)$$

$$I_{Nabk_{sl}} = F_{sl} \cdot G_{NaB} \cdot (V_m - E_{Na_{sl}}) \quad (S1.18)$$

$$I_{Nabk} = I_{Nabk_{junc}} + I_{Nabk_{sl}} \quad (S1.19)$$

I_{nak}: Na/K Pump Current

$$\sigma = \frac{e^{\frac{Nao}{67.3}-1}}{7} \quad (S1.20)$$

$$f_{nak} = \frac{1}{1+0.1248e^{-0.1V_m FoRT}} + 0.0365\sigma \cdot e^{-V_m \cdot FoRT} \quad (S1.21)$$

$$I_{nabk_{junc}} = \frac{F_{junc} \cdot \overline{I_{nak}} \cdot f_{nak}}{1+(\frac{Km_{Naip}}{Na_{junc}})^4} \frac{K_o}{K_o + Km_{K_o}} \quad (S1.22)$$

$$I_{nabk_{sl}} = \frac{F_{sl} \cdot \overline{I_{nak}} \cdot f_{nak}}{1+(\frac{Km_{Naip}}{Na_{sl}})^4} \frac{K_o}{K_o + Km_{K_o}} \quad (S1.23)$$

$$I_{nabk} = I_{nabk_{junc}} + I_{nabk_{sl}} \quad (S1.24)$$

I_{kr}: Rapidly Activating K Current

$$g_{kr} = 0.035\sqrt{\frac{K_o}{5.4}} \quad (S1.25)$$

$$x_{r_{ss}} = \frac{1}{1+e^{-\frac{V_m+10}{5}}} \quad (S1.26)$$

$$\tau_{xr} = \frac{550}{1+e^{-\frac{V_m+22}{9}}} \cdot \frac{6}{1+e^{\frac{V_m+11}{9}}} + \frac{230}{1+e^{\frac{V_m+40}{20}}} \quad (S1.27)$$

$$\dot{x_{kr}} = \frac{x_{r_{ss}} - x_{kr}}{\tau_{kr}} \quad (S1.28)$$

$$\tau_{kr} = \frac{550}{1+e^{\frac{V_m+74}{24}}} \quad (S1.29)$$

$$I_{kr} = g_{kr} \cdot x_{kr} \cdot r_{kr} \cdot (V_m - E_K) \quad (S1.30)$$

$I_k s$: Slowly Activating K Current.

$$xs_{ss} = \frac{1}{1+e^{-\frac{V_m+3.8}{14.25}}} \quad (S1.31)$$

$$\tau_{xs} = \frac{990.1}{1+e^{-\frac{V_m+2.436}{14.12}}} \quad (S1.32)$$

$$\dot{x_{ks}} = \frac{xs_{ss} - x_{ks}}{\tau_{ks}} \quad (S1.33)$$

$$I_{ks_{junc}} = F_{junc} \cdot g_{ks_{junc}} \cdot x_{ks}^2 \cdot (V_m - E_{Ks_{junc}}) \quad (S1.34)$$

$$I_{ks_{sl}} = F_{sl} \cdot g_{ks_{sl}} \cdot x_{ks}^2 \cdot (V_m - E_{Ks_{sl}}) \quad (S1.35)$$

$$I_{ks} = I_{ks_{junc}} + I_{ks_{sl}} \quad (S1.36)$$

I_{kp}: Plateau K current.

$$kp_{kp} = \frac{1}{1+e^{7.488-\frac{V_m}{5.98}}} \tag{S1.37}$$

$$I_{kp_{junc}} = F_{junc} \cdot g_{kp} \cdot kp_{kp} \cdot (V_m - E_K) \tag{S1.38}$$

$$I_{kp_{sl}} = F_{sl} \cdot g_{kp} \cdot kp_{kp} \cdot (V_m - E_K) \tag{S1.39}$$

$$I_{kp} = I_{kp_{junc}} + I_{kp_{sl}} \tag{S1.40}$$

I_{to}: Transient Outward K current

Slow Component

$$x_{to_{ss}} = \frac{1}{1+e^{-\frac{V_m-19.0}{13}}} \tag{S1.41}$$

$$y_{to_{ss}} = \frac{1}{1+e^{\frac{V_m+19.5}{5}}} \tag{S1.42}$$

$$\tau_{x_{to_{ss}}} = \frac{9}{1+e^{\frac{V_m+3.0}{15}}} + 0.5 \tag{S1.43}$$

$$\tau_{y_{to_{ss}}} = \frac{800}{1+e^{\frac{V_m+60.0}{10}}} + 30 \tag{S1.44}$$

$$\dot{x_{to_{ss}}} = \frac{x_{to_{ss}} - x_{to_s}}{\tau_{x_{to_s}}} \tag{S1.45}$$

$$\dot{y_{to_{ss}}} = \frac{y_{to_{ss}} - y_{to_s}}{\tau_{y_{to_s}}} \tag{S1.46}$$

$$I_{to_s} = G_{to_{Slow}} \cdot x_{to_{ss}} \cdot y_{to_{ss}} \cdot (V_m - E_K) \tag{S1.47}$$

Fast Component

$$\tau_{x_{to_f}} = 8.5e^{-(\frac{V_m+45}{50})^2} + 0.5 \tag{S1.48}$$

$$\tau_{y_{to_f}} = 85e^{-(\frac{V_m+40}{220})^2} + 7 \tag{S1.49}$$

$$\dot{x_{to_f}} = \frac{x_{to_{ss}} - x_{to_f}}{\tau_{x_{to_f}}} \tag{S1.50}$$

$$\dot{y_{to_f}} = \frac{y_{to_{ss}} - y_{to_f}}{\tau_{y_{to_f}}} \tag{S1.51}$$

$$I_{to_f} = G_{to_{Fast}} \cdot x_{to_f} \cdot y_{to_f} \cdot (V_m - E_K) \tag{S1.52}$$

$$I_{to} = I_{to_s} + I_{to_f} \tag{S1.53}$$

I_{K1}: Inward Rectifier K Current

$$a_{K1} = \frac{1.02}{1+e^{0.2385(V_m-E_K-59.215)}} \quad (S1.54)$$

$$b_{K1} = \frac{0.49124e^{0.08032(V_m+5.476-E_K)}+e^{0.06175(V_m-594.3-E_K)}}{1+e^{-0.5143(V_m+4.753-E_K)}} \quad (S1.55)$$

$$K1_{ss} = \frac{a_{K1}}{a_{K1}+b_{K1}} \quad (S1.56)$$

$$I_{K1} = 0.35\sqrt{\frac{K_0}{5.4}}\cdot K1_{ss}\cdot(V_m - E_K) \quad (S1.57)$$

I_{ClCa}: Ca-activated Cl Current

$$I_{ClCa_{junc}} = \frac{F_{junc}\cdot G_{ClCa}\cdot(V_m-E_{cl})}{1+\frac{Kd_{ClCa}}{Ca_j}} \quad (S1.58)$$

$$I_{ClCa_{sl}} = \frac{F_{sl}\cdot G_{ClCa}\cdot(V_m-E_{cl})}{1+\frac{Kd_{ClCa}}{Ca_sl}} \quad (S1.59)$$

$$I_{ClCa} = I_{ClCa_{junc}} + I_{ClCa_{sl}} \quad (S1.60)$$

I_{Clbk}: Background Cl Current

$$I_{Clbk} = G_{Cl_B}(V_m - E_{cl}) \quad (S1.61)$$

I_{Ca}: L-type Calcium Current

$$d_{ss} = \frac{1}{1+e^{-\frac{V_m+5}{6.0}}} \quad (S1.62)$$

$$\tau_d = d_{ss}\frac{1-e^{-\frac{V_m+5}{6.0}}}{0.035(V_m+5)} \quad (S1.63)$$

$$f_{ss} = \frac{1}{1+e^{\frac{V_m+35}{9}}} + \frac{0.6}{1+e^{\frac{50-V_m}{20}}} \quad (S1.64)$$

$$\tau_f = \frac{1}{0.0197e^{-0.0337(V_m+14.5)^2}+0.02} \quad (S1.65)$$

$$\dot{d} = \frac{d_{ss}-d}{\tau_d} \quad (S1.66)$$

$$\dot{f} = \frac{f_{ss}-f}{\tau_f} \quad (S1.67)$$

$$f_{Ca_{B_j}} = 1.7Ca_j(1 - f_{Ca_{B_j}}) - 11.9\times10^{-3}f_{Ca_{B_j}} \quad (S1.68)$$

$$f_{Ca_{B_{sl}}} = 1.7Ca_{sl}(1 - f_{Ca_{B_{sl}}}) - 11.9\times10^{-3}f_{Ca_{B_{sl}}} \quad (S1.69)$$

$$\overline{I_{ca_j}} = \frac{PCa\cdot 4(V_m\cdot Frdy\cdot FoRT)\cdot 0.341(Ca_j\cdot e^{2V_m\cdot FoRT}-Ca_o)}{e^{2V_m\cdot FoRT}-1} \quad (S1.70)$$

$$\overline{I_{ca_{sl}}} = \frac{PCa\cdot 4(V_m\cdot Frdy\cdot FoRT)\cdot 0.341(Ca_{sl}\cdot e^{2V_m\cdot FoRT}-Ca_o)}{e^{2V_m\cdot FoRT}-1} \quad (S1.71)$$

$$\overline{I_K} = p_K\frac{V_m\cdot Frdy\cdot FoRT\cdot 0.75(K_i\cdot e^{V_m\cdot FoRT}-K_o)}{e^{V_m\cdot FoRT}-1} \quad (S1.72)$$

$$\overline{I_{Na_j}} = p_N a \frac{V_m \cdot Frdy \cdot FoRT \cdot 0.75(Na_j \cdot e^{V_m \cdot FoRT} - Na_o)}{e^{V_m \cdot FoRT} - 1} \qquad (S1.73)$$

$$\overline{I_{na_{sl}}} = p_N a \frac{V_m \cdot Frdy \cdot FoRT \cdot 0.75(Na_{sl} \cdot e^{V_m \cdot FoRT} - Na_o)}{e^{V_m \cdot FoRT} - 1} \qquad (S1.74)$$

$$I_{Ca_{junc}} = F_{junc_{Ca_L}} \cdot \overline{I_{Ca_j}} \cdot d \cdot f \cdot (1 - f_{Ca_{B_j}}) \qquad (S1.75)$$

$$I_{Ca_{sl}} = F_{sl_{Ca_L}} \cdot \overline{I_{Ca_{sl}}} \cdot d \cdot f \cdot (1 - f_{Ca_{B_{sl}}}) \qquad (S1.76)$$

$$I_{Ca} = I_{Ca_{junc}} + I_{Ca_{sl}} \qquad (S1.77)$$

$$I_{Ca_K} = \overline{I_K} \cdot d \cdot f \cdot (F_{junc_{Ca_L}} \cdot (1 - f_{Ca_{B_j}}) + F_{sl_{Ca_L}} \cdot (1 - f_{Ca_{B_{sl}}}) \qquad (S1.78)$$

$$I_{Ca_{Na_{junc}}} = F_{junc_{Ca_L}} \cdot \overline{I_{Na_j}} \cdot d \cdot f \cdot (1 - f_{Ca_{B_j}}) \qquad (S1.79)$$

$$I_{Ca_{Na_{sl}}} = F_{sl_{Ca_L}} \cdot \overline{I_{Na_{sl}}} \cdot d \cdot f \cdot (1 - f_{Ca_{B_{sl}}}) \qquad (S1.80)$$

$$I_{Ca_{Na}} = I_{Ca_{Na_{junc}}} + I_{Ca_{Na_{sl}}} \qquad (S1.81)$$

$$I_{Ca_{tot}} = I_{Ca} + I_{Ca_K} + I_{Ca_{Na}} \qquad (S1.82)$$

$I_{Ca_{Na_{sl}}}$: Na/Ca Exchanger current

$$Ka_{junc} = \frac{1}{1 + (\frac{Kd_{act}}{Ca_j})^2} \qquad (S1.83)$$

$$Ka_{sl} = \frac{1}{1 + (\frac{Kd_{act}}{Ca_{sl}})^2} \qquad (S1.84)$$

$$s1_{junc} = e^{nu \cdot V_m \cdot FoRT} \cdot Na_j^3 \cdot Ca_o \qquad (S1.85)$$

$$s1_{sl} = e^{nu \cdot V_m \cdot FoRT} \cdot Na_{sl}^3 \cdot Ca_o \qquad (S1.86)$$

$$s2_{junc} = e^{(nu-1) \cdot V_m \cdot FoRT} \cdot Na_o^3 \cdot Ca_j \qquad (S1.87)$$

$$s2_{sl} = e^{(nu-1) \cdot V_m \cdot FoRT} \cdot Na_o^3 \cdot Ca_{sl} \qquad (S1.88)$$

$$\begin{aligned} s3_{junc} = {} & Km_{Ca_i} \cdot Na_o^3 \cdot (1 + (\frac{Na_j}{Km_{Na_i}})^3) + Km_{Na_o}^3 \cdot Ca_j \cdot (1 + \frac{Ca_j}{Km_{Ca_i}}) \\ & + Km_{Ca_o} \cdot Na_j^3 + Na_j^3 \cdot Ca_o + Na_o^3 \cdot Ca_j \end{aligned} \qquad (S1.89)$$

$$\begin{aligned} s3_{sl} = {} & Km_{Ca_{sl}} \cdot Na_o^3 \cdot (1 + (\frac{Na_{sl}}{Km_{Na_i}})^3) + Km_{Na_o}^3 \cdot Ca_{sl} \cdot (1 + \frac{Ca_{sl}}{Km_{Ca_i}}) \\ & + Km_{Ca_o} \cdot Na_{sl}^3 + Na_{sl}^3 \cdot Ca_o + Na_o^3 \cdot Ca_{sl} \end{aligned} \qquad (S1.90)$$

$$I_{ncx_{junc}} = \frac{F_{junc} \cdot \overline{I_{NCX}} \cdot Ka_{junc} \cdot (s1_{junc} - s2_{junc})}{s3_{junc} \cdot (1 + k_{sat} \cdot e^{nu-1}) \cdot V_m \cdot FoRT} \qquad (S1.91)$$

$$I_{ncx_{sl}} = \frac{F_{sl} \cdot \overline{I_{NCX}} \cdot Ka_{sl} \cdot (s1_{sl} - s2_{sl})}{s3_{sl} \cdot (1 + k_{sat} \cdot e^{nu-1}) \cdot V_m \cdot FoRT} \qquad (S1.92)$$

$$I_{ncx} = I_{ncx_{junc}} + I_{ncx_{sl}} \qquad (S1.93)$$

I_{pCa}: Sarcolemmal Ca pump Current

$$I_{pCa_{junc}} = \frac{F_{junc}\cdot\overline{I_{PMCA}}\cdot Ca_j^{1.6}}{Km_{P_{Ca}}^{1.6}+Ca_j^{1.6}} \quad (S1.94)$$

$$I_{pCa_{sl}} = \frac{F_{sl}\cdot\overline{I_{PMCA}}\cdot Ca_{sl}^{1.6}}{Km_{P_{Ca}}^{1.6}+Ca_{sl}^{1.6}} \quad (S1.95)$$

$$I_{pCa} = I_{pCa_{junc}} + I_{pCa_{sl}} \quad (S1.96)$$

I_{Cabk}: Background Ca Current

$$I_{Cabk_{junc}} = F_{junc}\cdot G_{Ca_B}\cdot(V_m - E_{Ca_{junc}}) \quad (S1.97)$$

$$I_{Cabk_{sl}} = F_{sl}\cdot G_{Ca_B}\cdot(V_m - E_{Ca_{sl}}) \quad (S1.98)$$

$$I_{Cabk} = I_{Cabk_{junc}} + I_{Cabk_{sl}} \quad (S1.99)$$

SR fluxes: Calcium Release, SR Ca pump, SR Ca leak

$$Max_{SR} = 15 \quad (S1.100)$$

$$Min_{SR} = 1 \quad (S1.101)$$

$$k_{Ca_{SR}} = Max_{SR} - \frac{Max_{SR}-Min_{SR}}{1+(\frac{ec50_{SR}}{Ca_{SR}})^{2.5}} \quad (S1.102)$$

$$ko_{SR_{Ca}} = \frac{ko_{Ca}}{k_{Ca_{SR}}} \quad (S1.103)$$

$$ki_{SR_{Ca}} = ki_{Ca}\cdot k_{Ca_{SR}} \quad (S1.104)$$

$$RI = 1 - Ry_{R_r} - Ry_{R_o} - Ry_{R_i} \quad (S1.105)$$

$$\dot{Ry}_{R_r} = (ki_m\cdot RI - ki_{SRCa}\cdot Ca_j\cdot Ry_{R_r}) - (ko_{SR_{Ca}}\cdot Ca_j^2\cdot Ry_{R_r} - ko_m\cdot Ry_{R_o}) \quad (S1.106)$$

$$\dot{Ry}_{R_o} = (ko_{SR_{Ca}}\cdot Ca_j^2\cdot Ry_{R_r} - ko_m\cdot Ry_{R_o}) - (ki_{SR_{Ca}}\cdot Ca_j\cdot Ry_{R_o} - ki_m\cdot Ry_{R_i}) \quad (S1.107)$$

$$\dot{Ry}_{R_i} = (ki_{SR_{Ca}}\cdot Ca_j\cdot Ry_{R_o} - ki_m\cdot Ry_{R_i}) - (ko_m\cdot Ry_{R_i} - ko_{SR_{Ca}}\cdot Ca_j^2\cdot RI) \quad (S1.108)$$

$$J_{SR_{Ca_{rel}}} = k_s\cdot Ry_{R_o}\cdot(Ca_{SR} - Ca_j) \quad (S1.109)$$

$$J_{SR_{Ca_{rel}}} = V_{max_{SR_{CaP}}}\cdot\frac{(\frac{Ca_i}{Km_f})^{kill_{SR_{CaP}}} - (\frac{Ca_{SR}}{Km_r})^{kill_{SR_{CaP}}}}{1+(\frac{Ca_i}{Km_f})^{kill_{SR_{CaP}}}+(\frac{Ca_{SR}}{Km_r})^{kill_{SR_{CaP}}}} \quad (S1.110)$$

$$J_{SR_{leak}} = 5.348\times10^{-6}(Ca_{SR} - Ca_j) \quad (S1.111)$$

Ion Homeostasis

Sodium Buffers

$$\dot{Na_{B_j}} = k_{on_{Na}} \cdot Na_j \cdot (B_{max_{Na_j}} - Na_{B_j}) - k_{off_{Na}} \cdot Na_{B_j} \tag{S1.112}$$

$$\dot{Na_{B_{sl}}} = k_{on_{Na}} \cdot Na_{sl} \cdot (B_{max_{Na_{sl}}} - Na_{B_{sl}}) - k_{off_{Na}} \cdot Na_{B_{sl}} \tag{S1.113}$$

Cytosolic Ca Buffers

$$\dot{T_n C_l} = k_{on_{T_n C_l}} \cdot Ca_i \cdot (B_{max_{T_n C_{low}}} - TnC_l) - k_{off_{T_n C_l}} \cdot T_n C_l \tag{S1.114}$$

$$T_n \dot{C}_{h_c} = k_{on_{T_n C_{h_{Ca}}}} \cdot Ca_i \cdot (B_{max_{T_n C_{high}}} - TnC_{h_c} - TnC_{h_m}) - k_{off_{T_n C_{h_{Ca}}}} \cdot T_n C_{h_c} \tag{S1.115}$$

$$T_n \dot{C}_{h_m} = k_{on_{T_n C_{h_{Mg}}}} \cdot Mg_i \cdot (B_{max_{T_n C_{high}}} - T_n C_{h_c} - T_n C_{h_m}) - k_{off_{T_n C_{h_{Mg}}}} \cdot T_n C_m \tag{S1.116}$$

$$\dot{CaM} = k_{on_{CaM}} \cdot Ca_i \cdot (B_{max_{CaM}} - CaM) - k_{off_{CaM}} \cdot CaM \tag{S1.117}$$

$$\dot{Myo_c} = k_{on_{myo_{Ca}}} \cdot Ca_i \cdot (B_{max_{myosin}} - Myo_c - Myo_m) - k_{off_{myo_{Ca}}} \cdot Myo_c \tag{S1.118}$$

$$\dot{Myo_m} = k_{on_{myo_{Mg}}} \cdot Mg_i \cdot (B_{max_{myosin}} - Myo_c - Myo_m) - k_{off_{myo_{Mg}}} \cdot Myo_m \tag{S1.119}$$

$$\dot{SRB} = k_{on_{sr}} \cdot Ca_i \cdot (B_{max_{SR}} - SRB) - k_{off_{sr}} \cdot SRB \tag{S1.120}$$

$$J_{Ca_{B_{cytosol}}} = \dot{T_n C_l} + T_n \dot{C}_{h_c} + T_n \dot{C}_{h_m} + \dot{CaM} + \dot{Myo_c} + \dot{Myo_m} + \dot{SRB} \tag{S1.121}$$

Junctional and SL Ca Buffers

$$\dot{SLL_j} = k_{on_{sl_l}} \cdot Ca_j \cdot (B_{max_{SL_{low_j}}} - SLL_j) - k_{off_{sl_l}} \cdot SLL_j \tag{S1.122}$$

$$\dot{SLL_{sl}} = k_{on_{sl_l}} \cdot Ca_{sl} \cdot (B_{max_{SL_{low_{sl}}}} - SLL_{sl}) - k_{off_{sl_l}} \cdot SLL_{sl} \tag{S1.123}$$

$$\dot{SLH_j} = k_{on_{sl_h}} \cdot Ca_j \cdot (B_{max_{SL_{high_j}}} - SLH_j) - k_{off_{sl_h}} \cdot SLH_j \tag{S1.124}$$

$$\dot{SLH_{sl}} = k_{on_{sl_h}} \cdot Ca_{sl} \cdot (B_{max_{SL_{high_{sl}}}} - SLH_{sl}) - k_{off_{sl_h}} \cdot SLH_{sl} \tag{S1.125}$$

$$J_{Ca_{B_{junction}}} = \dot{SLL_j} + \dot{SLH_j} \tag{S1.126}$$

$$J_{Ca_{B_{sl}}} = \dot{SLL_{sl}} + \dot{SLH_{sl}} \tag{S1.127}$$

SR Ca Buffer

$$\dot{Csqn_b} = k_{on_{csqn}} \cdot\cdot Ca_{SR} \cdot (B_{max_{csqn}} - Csqn_b) \cdot k_{off_{Caqn_b}} \cdot Csqn_b \qquad (S1.128)$$

Sodium Concentrations

$$I_{Na_{tot_{junc}}} = I_{Na_{junc}} + I_{Na_{bk_{junc}}} + 3I_{ncx_{junc}} + 3I_{nak_{junc}} + I_{Ca_{Na_{junc}}} \qquad (S1.129)$$

$$I_{Na_{tot_{sl}}} = I_{Na_{sl}} + I_{Na_{bk_{sl}}} + 3I_{ncx_{sl}} + 3I_{nak_{sl}} + I_{Ca_{Na_{sl}}} \qquad (S1.130)$$

$$\dot{Na_j} = -I_{Na_{tot_{junc}}} \cdot \frac{C_{mem}}{V_{junc} \cdot Frdy} + \frac{J_{na_{junc_{sl}}}}{V_{junc}} \cdot (Na_{sl} - Na_j) - \dot{Na_{B_j}} \qquad (S1.131)$$

$$\dot{Na_{sl}} = -I_{Na_{tot_{sl}}} \cdot \frac{C_{mem}}{V_{sl} \cdot Frdy} + \frac{J_{na_{junc_{sl}}}}{V_{sl}} \cdot (Na_j - Na_{sl}) + \frac{J_{na_{slmyo}}}{V_{sl}} \cdot (Na_i - Na_{sl}) - \dot{Na_{B_{sl}}} \qquad (S1.132)$$

$$\dot{Na_i} = \frac{J_{Na_{slmyo}}}{V_{myo}} \cdot (Na_{sl} - Na_i) \qquad (S1.133)$$

Potassium Concentration

$$I_{K_{tot}} = I_{to} + I_{kr} + I_{ks} + I_{K1} - 2I_{nak} + I_{Cak} + I_{kp} \qquad (S1.134)$$

$$\dot{K_i} = 0 \qquad (S1.135)$$

Calcium Concentrations

$$I_{Ca_{tot_{junc}}} = I_{Ca_{junc}} + I_{Ca_{bk_{junction}}} + I_{pCa_{junc}} - 2I_{ncx_{junc}} \qquad (S1.136)$$

$$I_{Ca_{tot_{sl}}} = I_{Ca_{sl}} + I_{Ca_{bk_{sl}}} + I_{pCa_{sl}} - 2I_{ncx_{sl}} \qquad (S1.137)$$

$$\dot{Ca_j} = -I_{Ca_{tot_{junc}}} \cdot \frac{C_{mem}}{V_{junc} \cdot 2Frdy} + \frac{J_{Ca_{juncsl}}}{V_{junc}} \cdot (Ca_{sl} - Ca_j) - I_{Ca_{B_{junction}}} + J_{SR_{Ca_{rel}}} \cdot \frac{V_{sr}}{V_{junc}} + J_{SR_{leak}} \cdot \frac{V_{myo}}{V_{junc}} \qquad (S1.138)$$

$$\dot{Ca_{sl}} = -I_{Ca_{tot_{sl}}} \cdot \frac{C_{mem}}{V_{sl} \cdot 2Frdy} + \frac{J_{Ca_{juncsl}}}{V_{sl}} \cdot (Ca_{s1} - Ca_{sl}) + \frac{V_{Ca_{slmyo}}}{V_{sl}} \cdot (Ca_i - Ca_{sl}) + J_{Ca_{B_{sl}}} \qquad (S1.139)$$

$$\dot{Ca_i} = -J_{SR_{Ca}} \cdot \frac{V_{sr}}{V_{myo}} - Ca_{B_{cytoslo}} + \frac{J_{Ca_{slmyo}}}{V_{myo}} \cdot (Ca_{sl} - Ca_i) \qquad (S1.140)$$

$$\dot{Ca_{SR}} = J_{ser_{Ca}} - (J_{SR_{leak}} \cdot \frac{V_{myo}}{V_{sr}} + J_{SR_{Ca_{rel}}}) - \dot{Csqn_b} \qquad (S1.141)$$

Membrane Potential

$$I_{Na_{tot}} = I_{Na_{tot_{junc}}} + I_{Na_{tot_{sl}}} \quad (S1.142)$$

$$I_{Cl_{tot}} = I_{ClCa} + I_{Clbk} \quad (S1.143)$$

$$I_{Ca_{tot}} = I_{Ca_{tot_{junc}}} + I_{Ca_{tot_{sl}}} \quad (S1.144)$$

$$I_{tot} = I_{Na_{tot}} + I_{Cl_{to}} + I_{Ca_{tot}} + I_{K_{tot}} \quad (S1.145)$$

$$\dot{V_m} = -(I_{tot} - I_{app}) \quad (S1.146)$$

Nernst Potentials

$$E_{Na_{junc}} = \frac{1}{FoRT} \cdot log(\frac{Na_o}{Na_j}) \quad (S1.147)$$

$$E_{Na_{sl}} = \frac{1}{FoRT} \cdot log(\frac{Na_o}{Na_{sl}}) \quad (S1.148)$$

$$E_K = \frac{1}{FoRT} \cdot log(\frac{K_o}{K_i}) \quad (S1.149)$$

$$E_{Ks_{junc}} = \frac{1}{FoRT} \cdot log(\frac{K_o + pnakNa_o}{K_i + pnakNa_j}) \quad (S1.150)$$

$$E_{Ks_{sl}} = \frac{1}{FoRT} \cdot log(\frac{K_o + pnakNa_o}{K_i + pnakNa_{sl}}) \quad (S1.151)$$

$$E_{Ca_{junc}} = \frac{1}{2FoRT} \cdot log(\frac{Ca_o}{Ca_j}) \quad (S1.152)$$

$$E_{Ca_{sl}} = \frac{1}{2FoRT} \cdot log(\frac{Ca_o}{Ca_{sl}}) \quad (S1.153)$$

$$E_{Cl} = \frac{1}{FoRT} \cdot log(\frac{Cl_i}{Cl_o}) \quad (S1.154)$$
