# Supplementary material for: A mathematical model for active contraction in healthy and failing myocytes and left ventricles
Source: PLoS One. 2017 Apr 13;12(4):e0174834. doi: 10.1371/journal.pone.0174834 (PMC5391010; doi:10.1371/journal.pone.0174834)
Supplement: S2 Appendix — (PDF) [file pone.0174834.s002.pdf]

S2 Appendix: Parameters of the original GPB model

**Physical**					
Parameter	Numerical value	Unit	Parameter	Numerical value	Unit
R	8314	$[J/(kmol \cdot K)]$	$Frdy$	96485	$[C/mol]$
$Temp$	310	$[K]$	$FoRT$	3597.59	$[1/mV]$
C_{mem}	1.3810×10^{-10}	$[F]$			
**Fractional**					
Parameter	Numerical value	Unit	Parameter	Numerical value	Unit
F_{junc}	0.11	$[-]$	F_{sl}	$1 - F_{junc}$	$[-]$
$F_{junc_{Ca_L}}$	0.9	$[-]$	$F_{sl_{Ca_L}}$	$1 - F_{junc_{Ca_L}}$	$[-]$
**Environmental Parameters**					
Parameter	Numerical value	Unit	Parameter	Numerical value	Unit
$cellLength$	100	$[\mu m]$	$juncLength$	160×10^{-3}	$[\mu m]$
$cellRadius$	10.25	$[\mu m]$	$juncRadius$	15×10^{-3}	$[\mu m]$
$distSLcyto$	0.45	$[\mu m]$	$distJuncSL$	0.5	$[\mu m]$
$DcaJuncSL$	1.2205×10^{-5}	$[cm^2/sec]$	$DcaSLcyto$	2.8914×10^{-6}	$[cm^2/sec]$
$DnaJuncSL$	2.7121×10^{-7}	$[cm^2/sec]$	$DnaSLcyto$	1.2722×10^{-6}	$[cm^2/sec]$
V_{cell}	33×10^{-12}	$[L]$	V_{myo}	$0.65V_{cell}$	$[\mu m^2]$
V_{sr}	$0.035V_{cell}$	$[\mu m^2]$	V_{sl}	$0.02V_{cell}$	$[\mu m^2]$
V_{junc}	$5.39\times10^{-4}VV_{cell}$	$[\mu m^2]$	SA_{junc}	303.855	$[\mu m^2]$
SA_sl	6440.26	$[\mu m^2]$	$J_{Ca_{juncsl}}$	8.2413×10^{-13}	$[L/ms]$
$J_{Ca_{slmyo}}$	3.7243×10^{-12}	$[L/ms]$	$J_{na_{juncsl}}$	1.83128×10^{-14}	$[L/ms]$
$J_{na_{slmyo}}$	1.63863×10^{-12}	$[L/ms]$			
**Ion**					
Parameter	Numerical value	Unit	Parameter	Numerical value	Unit
C_i	15	$[mM]$	Mg_i	1	$[mM]$
K_i	120	$[mM]$	Cl_o	150	$[mM]$
K_o	5.4	$[mM]$	Na_o	140	$[mM]$
Ca_o	1.8	$[mM]$			
**Na transport**					
Parameter	Numerical value	Unit	Parameter	Numerical value	Unit
G_{Na}	23	$[mS/\mu M]$	G_{Na_B}	0.597×10^{-3}	$[mS/\mu M]$
I_{Na_K}	1.8	$[A/F]$	$K_{m_{Naip}}$	11	$[mM]$
$K_{m_{K_o}}$	1.5	$[mM]$			
**K**					
Parameter	Numerical value	Unit	Parameter	Numerical value	Unit
P_{Na_K}	0.01833	$[-]$	g_{k_p}	0.002	$[mS/\mu F]$
$P_{ks_{junc}}$	0.0035	$[mS/\mu F]$	$g_{ks_{sl}}$	0.0035	$[mS/\mu F]$
$[EPI]G_{to_{Slow}}$	0.0156	$[mS/\mu F]$	$[Endo]G_{to_{Slow}}$	0.0376	$[mS/\mu F]$
$[EPI]G_{to_{Fast}}$	0.1144	$[mS/\mu F]$	$[Endo]G_{to_{Fast}}$	0.0014	$[mS/\mu F]$
**Cl Currents**					
Parameter	Numerical value	Unit	Parameter	Numerical value	Unit
$G_{Cl_{Ca}}$	0.0548125	$[mS/\mu F]$	$Kd_{Cl_{Ca}}$	100×10^{-3}	$[mS/M]$
G_{Cl_B}	9×100^{-3}	$[mS/\mu F]$			

**Ca transport**					
Parameter	Numerical value	Unit	Parameter	Numerical value	Unit
p_{Na}	3.375×10^{-9}	$[cm/sec]$	p_{Ca}	1.215×10^{-4}	$[cm/sec]$
p_K	6.075×10^{-8}	$[cm/sec]$	$\overline{I_{NCX}}$	4.5	$[A/F]$
Km_{Ca_i}	3.59×10^{-3}	$[mM]$	Km_{Ca_o}	1.3	$[mM]$
Km_{Na_i}	12.29	$[mM]$	Km_{Na_o}	87.5	$[mM]$
k_{sat}	0.32	$[-]$	nu	0.27	$[-]$
Kd_{act}	0.150×10^{-3}	$[mM]$	$\overline{I_{PMCA}}$	0.0673	$[A/F]$
$Km_{P_{Ca}}$	0.5×10^{-3}	$[mM]$	G_{Ca_B}	5.513×10^{-4}	$[A/F]$
**SR Ca fluxes**					
Parameter	Numerical value	Unit	Parameter	Numerical value	Unit
$Vmax_{SR_{Ca_P}}$	5.3114×10^{-3}	$[mM/ms]$	Km_f	0.2463×10^{-3}	$[mM]$
Km_r	1.7	$[mM]$	$hill_{SR_{Ca_P}}$	1.787	$[-]$
ks	25	$[ms^{-1}]$	Ko_{Ca}	10	$[ms^{-1}mM^{2}]$
Ko_m	0.06	$[ms^{-1}]$	Ki_{Ca}	0.5	$[ms^{-1}mM^{-1}]$
Ki_m	0.005	$[ms^{-1}]$	$ec50SR$	0.45	$[mM]$
**Buffering**					
Parameter	Numerical value	Unit	Parameter	Numerical value	Unit
$B_{max_{Na_j}}$	7.561	$[mM]$	$B_{max_{Na_{sl}}}$	1.65	$[mM]$
$k_{off_{Na}}$	1×10^{-3}	$[ms^{-1}]$	$k_{on_{Na}}$	0.1×10^{-3}	$[ms^{-1}mM^{-1}]$
$B_{max_{TnC_{low}}}$	70×10^{-3}	$[mM]$	$B_{max_{TnC_{high}}}$	140×10^{-3}	$[mM]$
$k_{off_{TnC_l}}$	19.6×10^{-3}	$[ms^{-1}]$	$k_{off_{TnC_{h_{Ca}}}}$	0.032×10^{-3}	$[ms^{-1}]$
$k_{on_{TnC_l}}$	32.7	$[ms^{-1}mM^{-1}]$	$k_{on_{TnC_{h_{Ca}}}}$	2.37	$[ms^{-1}mM^{-1}]$
$k_{off_{TnC_{h_{Mg}}}}$	3.33×10^{-3}	$[ms^{-1}]$	$k_{on_{TnC_{h_{Mg}}}}$	3×10^{-3}	$[ms^{-1}mM^{-1}]$
$B_{max_{CaM}}$	24×10^{-3}	$[mM]$	$B_{max_{myosin}}$	140×10^{-3}	$[mM]$
$k_{off_{CaM}}$	238×10^{-3}	$[ms^{-1}]$	$k_{off_{myo_{Ca}}}$	0.46×10^{-3}	$[ms^{-1}]$
$k_{on_{CaM}}$	34	$[ms^{-1}mM^{-1}]$	$k_{on_{myo_{Ca}}}$	13.8	$[ms^{-1}mM^{-1}]$
$k_{off_{myo_{Mg}}}$	0.057×10^{-3}	$[ms^{-1}]$	$k_{on_{myo_{Mg}}}$	0.0157	$[ms^{-1}mM^{-1}]$
$B_{max_{SL_{low_{sl}}}}$	$37.4\times10^{-3}\cdot\frac{V_{myo}}{V_{sl}}$	$[mM]$	$B_{max_{SL_{low_j}}}$	$4.6\times10^{-4}\cdot\frac{V_{myo}}{V_{junc}}$	$[mM]$
$k_{off_{sl_l}}$	1300×10^{-3}	$[ms^{-1}]$	$k_{on_{sl_l}}$	100	$[ms^{-1}mM^{-1}]$
$B_{max_{SL_{high_{sl}}}}$	$13.4\times10^{-3}\cdot\frac{V_{myo}}{V_{sl}}$	$[mM]$	$B_{max_{SL_{high_j}}}$	$1.65\times10^{-4}\cdot\frac{V_{myo}}{V_{junc}}$	$[mM]$
$k_{off_{sl_h}}$	30×10^{-3}	$[ms^{-1}]$	$k_{on_{sl_h}}$	100	$[ms^{-1}mM^{-1}]$
$B_{max_{SR}}$	17.1×10^{-3}	$[mM]$	$B_{max_{Csqn}}$	$140\times10^{-3}\cdot\frac{V_{myo}}{V_{sr}}$	$[mM]$
$k_{off_{SR}}$	60×10^{-3}	$[ms^{-1}]$	$k_{off_{Csqn}}$	65	$[ms^{-1}]$
$k_{on_{SR}}$	100	$[ms^{-1}mM^{-1}]$	$k_{on_{Csqn}}$	100	$[ms^{-1}mM^{-1}]$
