# Supplementary material for: A mathematical model for active contraction in healthy and failing myocytes and left ventricles
Source: PLoS One. 2017 Apr 13;12(4):e0174834. doi: 10.1371/journal.pone.0174834 (PMC5391010; doi:10.1371/journal.pone.0174834)
Supplement: S3 Appendix — The modified GPB model is formulated by introducing the impact factors (see Table 1) to the original GPB model. (PDF) [file pone.0174834.s003.pdf]

S3 Appendix: The modified GPB model

For the GPB model

$$I_{Nabk} = f_{Nabk} \cdot (I_{Nabk_{junc}} + I_{Nabk_{sl}}) \quad (S3.1)$$

$$I_{nak} = f_{Nak} \cdot (I_{nak_{junc}} + I_{nak_{sl}}) \quad (S3.2)$$

$$I_{Ki} = f_{Ki} \cdot 0.35 \sqrt{\frac{K_0}{5.4}} \cdot K1_{ss} \cdot (V_m - E_K) \quad (S3.3)$$

$$I_{ncx} = f_{ncx} \cdot (I_{ncx_{junc}} + I_{ncx_{sl}}) \quad (S3.4)$$

$$I_{Cabk} = f_{Cabk} \cdot (I_{Cabk_{junc}} + I_{Cabk_{sl}}) \quad (S3.5)$$

$$J_{SR_{Ca_{rel}}} = f_{SRca} \cdot V_{max_{SR_{CaP}}} \cdot \frac{(\frac{Ca_i}{Km_f})^{kill_{SR_{CaP}}} - (\frac{Ca_{SR}}{Km_r})^{kill_{SR_{CaP}}}}{1 + (\frac{Ca_i}{Km_f})^{kill_{SR_{CaP}}} + (\frac{Ca_{SR}}{Km_r})^{kill_{SR_{CaP}}}} \quad (S3.6)$$

$$J_{SR_{leak}} = f_{SRleak} \cdot 5.348 \times 10^{-6} \cdot (Ca_{SR} - Ca_j) \quad (S3.7)$$

$$EC50SR = f_{EC50SR} \cdot EC50SR \quad (S3.8)$$

For the late sodium current

$$\tau_{hl} = f_{hl} \cdot \tau_{hl} \quad (S3.9)$$

$$I_{NaL_{junc}} = f_{NaL} \cdot F_{junc} \cdot g_{Nal} \cdot m_L^3 \cdot h_L \cdot (V_m - E_{junc}) \quad (S3.10)$$

$$I_{NaL_{sl}} = f_{NaL} \cdot F_{sl} \cdot g_{Nal} \cdot m_L^3 \cdot h_L \cdot (V_m - E_{sl}) \quad (S3.11)$$
