# Supplementary material for: A mathematical model for active contraction in healthy and failing myocytes and left ventricles
Source: PLoS One. 2017 Apr 13;12(4):e0174834. doi: 10.1371/journal.pone.0174834 (PMC5391010; doi:10.1371/journal.pone.0174834)
Supplement: S4 Appendix — (PDF) [file pone.0174834.s004.pdf]

S4 Appendix: The NHS myofilament model

By using fading memory model and the Hill equation, the active contraction force T can be defined by

$$T = \begin{cases} T_0 \cdot \frac{1+a\sum_{i=1}^{3} Q_i}{1-\sum_{i=1}^{3} Q_i} & \sum_{i=1}^{3} Q_i < 0 \\ T_0 \cdot \frac{1+(1+2a)\sum_{i=1}^{3} Q_i}{1+\sum_{i=1}^{3} Q_i} & \sum_{i=1}^{3} Q_i > 0 \end{cases} \tag{S4.1}$$

where T_0 is the isometric tension, a is a measure of the curvature of the force-velocity, Q_i is defined by

$$\frac{dQ_i}{dt} = A_i \frac{d\lambda_i}{dt} - \alpha_i Q_i \tag{S4.2}$$

where A_1, A_2, A_3, α_1, α_2 and α_3 are model parameters.

The isometric tension T_0, as a function of myocardium fibre stretch λ_f and tropomyosin described by z, describes the fraction of actin sties. Because of equality $\frac{T_0}{T_{0Max}} = \frac{z}{z_{Max}}$ and the following equation

$$T_{0Max} = T_{ref}(1 + \beta_0(\lambda - 1)), \tag{S4.3}$$

T_0 can be defined by

$$T_0 = T_{ref} \cdot (1 + \beta_0(\lambda - 1)) \cdot \frac{z}{z_{Max}} \tag{S4.4}$$

in which T_{ref} is the maximum tension at resting myocardium fibre stretch, β_0 is the slope of the $\lambda - T_{0Max}$ relationship. z_{Max} is the fraction of tropomyosin at full activation. z_{Max} can be defined by

$$z_{Max} = \frac{\frac{\alpha_0}{[Ca^{2+}]_{tr_{half}}^{n}} - \frac{\alpha_{r2} z_p^{n_r}}{z_p^{n_r} + K_z^{n_r}}(1 - \frac{n_r K_z^{n_r}}{z_p^{n_r} + K_z^{n_r}})}{\alpha_{r1} + \frac{\alpha_{r2} z_p^{n_r - 1} K_z^{n_r}}{(z_p^{n_r} + K_z^{n_r})^2} + \frac{\alpha_0}{[Ca^{2+}]_{tr_{half}}^{n}}} \tag{S4.5}$$

in which α_0, α_{r1}, α_{r2}, z_p, n_r, n and K_z are model parameters, $[Ca^{2+}]_{tr_{max}}$ is the maximum concentration of Ca^{2+} bound to troponin, $[Ca^{2+}]_{tr_{half}}$ is the concentration of Ca^{2+} bound to troponin at semi activation. $[Ca^{2+}]_{tr_{half}}$ can be defined by

$$[Ca^{2+}]_{tr_{half}} = \frac{[Ca^{2+}]_{tr_{max}} [Ca^{2+}]_{half}}{[Ca^{2+}]_{half} + \frac{k_{off_{ref}}}{k_{on}}(1 - \frac{1+\beta_0(\lambda_f - 1)}{2\gamma_0})} \tag{S4.6}$$

in which $[Ca^{2+}]_{half} = [Ca^{2+}]_{half_{ref}}(1 + \beta_1(\lambda_f - 1))$, β_0, β_1, γ_0, and $[Ca^{2+}]_{half_{ref}}$ are model parameters.

The tropomyosin described by z satisfies the equation defined by

$$\frac{dz}{dt} = \alpha_0 (\frac{[Ca^{2+}]_{tr}}{[Ca^{2+}]_{tr_{half}}})^n (1 - z) - \alpha_{r1} z - \alpha_{r2} \frac{z^{n_r}}{z^{n_r} + K_z^{n_r}} \tag{S4.7}$$

By using the intracellular concentration of Ca^{2+}, we can obtain the concentration of Ca^{2+} bound to troponin

$$\frac{d[Ca^{2+}]_{tr}}{dt} = k_{on} [Ca]_i^{2+} ([Ca^{2+}]_{tr_{max}} - [Ca^{2+}]_{tr}) - k_{off} [Ca^2+]_{tr} \tag{S4.8}$$

in which k_{on} is the binding rate, $k_{off} = k_{off_{ref}}(1 - \frac{T}{\gamma_0 T_{ref}})$ is unbinding rate, and $k_{off_{ref}}$ is the unbinding rate in the absence of tension. Finally, we can calculate the active tension $T(\mathbf{X}, t)$ using the equations mentioned above. The model parameters are list in the following table.

The parameters of NHS model								
Parameter	Value	Unit	Parameter	Value	Unit	Parameter	Value	Unit
a	0.35	$[-1]$	α_1	30	s^{-1}	α_2	130	s^{-1}
α_3	625	s^{-1}	A_1	-29	$[-]$	A_2	138	$[-]$
A_3	129	$[-]$	β_0	4.9	$[-]$	β_1	-4.0	$[-]$
Z_p	0.85	$[-]$	K_z	0.15	$[-]$	n_r	3	$[-]$
γ_0	2.0	$[-]$	$k_{off_{ref}}$	200	s^{-1}	k_{on}	100	μM^{-1}
T_{ref}	56.2	$[-]$	$[Ca^{2+}]_{half_{ref}}$	1.05	μM	$[Ca^{2+}]_{tr_{max}}$	70	μM
