# Supplementary material for: A mathematical model for active contraction in healthy and failing myocytes and left ventricles
Source: PLoS One. 2017 Apr 13;12(4):e0174834. doi: 10.1371/journal.pone.0174834 (PMC5391010; doi:10.1371/journal.pone.0174834)
Supplement: S1 Table — Comparisons are made between the modelling results from healthy cases and published experimental and numerical studies at organ and cellular level. (PDF) [file pone.0174834.s005.pdf]

S1 Table: Comparisons between the healthy cases and other studies

The organ level

	End-diastolic volume (mL)	End-systolic volume (mL)	Ejection fraction(%)	Myofibre strain
**our model**	145	61	57.55	-0.236 ± 0.0371
Lorenz et al [1]	121 ± 34	40 ± 14	67 ± 5	-
Moore et al [2]	-	-	-	-0.23 ± 0.04
Aleksander et al [3]	146.2 ± 20.8	51.0 ± 9.5	63.6 ± 5.7	-
Augustine et al [4]	-	-	63.88 ± 5.07	-0.21 ± 0.03
Neizel et al [5]	-	-	-	-0.222 ± 0.029
Mangion et al [6]	130.53 ± 20.57	47.87 ± 11.22	63.6 ± 5	-0.187 ± 0.022
Mangion et al [7]	130.39 ± 21.02	47.99 ± 12.28	63.6 ± 5.2	-0.28 ± 0.048

The cellular level

Action Potential

	APD_{90}(ms)	Amplitude	Resting potential(mV)	Maximal upstroke velocity(V/s)
**our model**	270.19	123.36	-80.55	370.70
Li et al [8]	271 ± 13	132	-83 ± 3	-
Li et al [9]	281 ± 21	100	-	-
Näbauer et al [10]	-	135	-79.5 ± 0.48	-
Drouin et al [11]	351 ± 14	-	-	228 ± 11
Péréon et al [12]	432 ± 19	106 ± 3	-86 ± 1	446 ± 46
Tusscher et al [13]	-	109	-87.3	288
Cai et al [14]	234.06	99.88	-85	65.59

Intracellular CaT

	Peak(μM)	Amplitude	Time to peak of CaT(s)	Maximal upstroke velocity(μM/s)
**our model**	0.9752	0.87	0.039	139.54
Hunter et al [15]	1.0	0.99	0.06	-
Tusscher et al [13]	1.0	0.93	-	-

References

1. Lorenz C H, Walker E S, Morgan V L, et al. Normal human right and left ventricular mass, systolic function, and gender differences by cine magnetic resonance imaging. Journal of Cardiovascular Magnetic Resonance, 1999;1(1):7-21.

2. Moore C C, Lugoolivieri C H, Mcveigh E R, et al. Three-dimensional Systolic Strain Patterns in the Normal Human Left Ventricle: Characterization with Tagged MR Imaging1. Radiology, 2000;214(2):453-66.

3. Aleksander K, Rodrigo F J, Stefan O, et al. Quantification of biventricular myocardial function using cardiac magnetic resonance feature tracking, endocardial border delineation and echocardiographic speckle tracking in patients with repaired tetralogy of fallot and healthy controls. Journal of Cardiovascular Magnetic Resonance, 2012;14(1):32.

4. Augustine D, Lewandowski A J, Lazdam M, et al. Global and regional left ventricular myocardial deformation measures by magnetic resonance feature tracking in healthy volunteers: comparison with tagging and relevance of gender. Journal of Cardiovascular Magnetic Resonance, 2013;15(1):1-10.

5. Neizel M, Lossnitzer D G, Schaufele T, et al. Strain-encoded (SENC) magnetic resonance imaging to evaluate regional heterogeneity of myocardial strain in healthy volunteers: Comparison with conventional tagging. Journal of Magnetic Resonance Imaging, 2008;29(1):99-105.

6. Mangion K, Clerfond G, Mccomb C, et al. Myocardial strain in healthy adults across a broad age range as revealed by cardiac magnetic resonance imaging at 1.5 and 3.0T: Associations of myocardial strain with myocardial region, age, and sex. Journal of Magnetic Resonance Imaging, 2016;44(5):1197-1205.

7. Mangion K, Gao H, Mccomb C, et al. A Novel Method for Estimating Myocardial Strain: Assessment of Deformation Tracking Against Reference Magnetic Resonance Methods in Healthy Volunteers. Scientific Reports, 2016;6:38774.

8. Li G R, Feng J, Yue L, et al. Transmural heterogeneity of action potentials and Ito1 in myocytes isolated from the human right ventricle. American Journal of Physiology, 1998;275(2):369-77.

9. Li G R, Yang B, Feng J, et al. Transmembrane ICa contributes to rate-dependent changes of action potentials in human ventricular myocytes. American Journal of Physiology, 1999;276(1 Pt 2):98-106.

10. Näbauer M, Beuckelmann D J, Uberfuhr P, et al. Regional differences in current density and rate-dependent properties of the transient outward current in subepicardial and subendocardial myocytes of human left ventricle. Circulation, 1996;93(1):168-77.

11. Drouin E, Lande G, Charpentier F. Amiodarone reduces transmural heterogeneity of repolarization in the human heart. Journal of the American College of Cardiology, 1998;32(4):1063.

12. Péréon Y, Demolombe S, Baró I, et al. Differential expression of KvLQT1 isoforms across the human ventricular wall. American Journal of Physiology Heart & Circulatory Physiology, 2000;278(6):1908-15.

13. ten Tusscher KH1, Noble D, Noble PJ, Panfilov AV. A model for human ventricular tissue. American Journal of Physiology Heart & Circulatory Physiology, 2004;286(286):1573-89.

14. Cai L, et al. Multi-scale modelling of the human left ventricle (in Chinese). SCIENTIA SINICA: Physica, Mechanica & Astronomica, 2015;2:36-45.

15. Hunter PJ, McCulloch AD, ter Keurs HE. Modelling the mechanical properties of cardiac muscle. Prog Biophys Mol Biol, 1998;69(2–3):289–331.
